# Defective expansion and function of memory like natural killer cells in HIV+ individuals with latent tuberculosis infection

**DOI:** 10.1371/journal.pone.0257185

**Published:** 2021-09-13

**Authors:** Kamakshi Prudhula Devalraju, Venkata Sanjeev Kumar Neela, Siva Sai Krovvidi, Ramakrishna Vankayalapati, Vijaya Lakshmi Valluri

**Affiliations:** 1 Immunology and Molecular Biology Division, Blue Peter Public Health and Research Centre, LEPRA Society, Cherlapally, Hyderabad, Telangana, India; 2 Department of Biotechnology, Sreenidhi Institute of Science and Technology, Yamnampet, Ghatkesar, Hyderabad, Telangana, India; 3 Department of Pulmonary Immunology, Center for Biomedical Research, The University of Texas Health Center at Tyler, Texas, TX, United States of America; Rutgers Biomedical and Health Sciences, UNITED STATES

## Abstract

**Purpose:**

Tuberculosis (TB) is the leading cause of infectious disease related mortality, and only 10% of the infected individuals develop active disease. The likelihood of progression of latent tuberculosis infection (LTBI) to active TB disease is high in HIV infected individuals. Identification of HIV+ individuals at risk would allow treating targeted population, facilitating completion of therapy for LTBI and prevention of TB development. NK cells have an important role in T cell independent immunity against TB, but the exact role of NK cell subsets in LTBI and HIV is not well characterized.

**Methods:**

In this study, we compared the expansion and function of memory like NK cells from HIV-LTBI+ individuals and treatment naïve HIV+LTBI+ patients in response to Mtb antigens ESAT-6 and CFP-10.

**Results:**

In freshly isolated PBMCs, percentages of CD3^-^CD56^+^ NK cells were similar in HIV+LTBI+ patients and healthy HIV-LTBI+ individuals. However, percentages of CD3^-^CD56^+^CD16^+^ NK cells were higher in healthy HIV-LTBI+ individuals compared to HIV+LTBI+ patients. HIV infection also inhibited the expansion of memory like NK cells, production of IL-32α, IL-15 and IFN-γ in response to Mtb antigens in LTBI+ individuals.

**Conclusion:**

We studied phenotypic, functional subsets and activation of memory like-NK cells during HIV infection and LTBI. We observed that HIV+LTBI+ patients demonstrated suboptimal NK cell and monocyte interactions in response to Mtb, leading to reduced IL-15, IFN-γ and granzyme B and increased CCL5 production. Our study highlights the effect of HIV and LTBI on modulation of NK cell activity to understand their role in development of interventions to prevent progression to TB in high risk individuals.

## 1. Introduction

*Mycobacterium tuberculosis* (Mtb) kills about 1.5 million individuals each year globally [1]. 10% of individuals with latent tuberculosis infection (LTBI), are at risk of developing active TB. Human Immunodeficiency Virus (HIV) infection is a major contributor to the risk of progression of LTBI to active TB disease [2, 3]. TB is the most common cause of death in HIV patients, contributing more than half a million deaths annually among HIV-TB coinfected patients (www.who.int/tb/areas-of-work/tb-hiv/en/). Identification of HIV+ individuals with LTBI who are at increased risk would allow targeted treatment to prevent development of active TB. To identify these individuals, it is important to pinpoint the nature of defects in immune responses that lead to development of active TB in HIV+LTBI+ patients.

Innate defenses play an important role against TB and HIV infections. Natural Killer (NK) cells are multifunctional CD3^-^CD56^+^ lymphocytes and important mediators of innate immune responses playing a key role in clearance of viruses and other intracellular pathogens [4, 5]. Recent studies suggest that NK cells are heterogeneous, distinguish antigens and differentiate into memory phenotype to protect against pathogens [6, 7]. NK cells are made up of phenotypically and functionally distinct subsets (cytolytic CD3^-^CD56^dim^CD16^+^ and IFN-γ producing CD3^-^CD56^bright^CD16^-^). Expression of maturation markers like CD27, CD11b and KLRG1 (Killer cell Lectin-like Receptor G1), separate individual NK cell subpopulations based on responsiveness, migratory capacity and anti-tumour activity [8, 9]. CD27 expressing NK cells are sub-categorised into cytokine producing CD27^high^ and CD27^low^ populations, that help in formation of memory T cells [10]. CD27 deficient mice have demonstrated defects in differentiation into effector and memory T cells [10, 11]. In our previous studies, we demonstrated that IL-21 dependent expansion of memory-like NK cells is crucial for inducing protective immunity against Mtb post BCG vaccination in humans, as well as mice [10].

HIV infection alters NK cell homeostasis and hampers their antiviral effector functions [12–14]. HIV infection also induces changes in the expression of activating and inhibitory receptors on NK cells there by impairing effector functions like cytotoxicity, cytokine production as well as ADCC responses [15, 16]. In HIV infected individuals, NK cells secrete cytokines that minimize viral entry and replication [17]. NK cells also have an important role in T cell independent immunity against HIV and LTBI [18, 19]. However, the exact role of NK subsets during latent TB infection and HIV was not addressed before. In this study we determined the distribution of NK cell subsets and their cytokine profile in freshly isolated and ESAT-6 and CFP-10 cultured PBMCs of HIV-LTBI+ individuals and treatment naïve HIV+LTBI+ patients.

## 2. Materials and methods

### 2.1 Study subjects

#### 2.1.1 HIV positive individuals

Hundred patients sero-positive to HIV diagnosed primarily by Combs (Arkray healthcare Pvt.Ltd) confirmed by Trispot and SD bioline tests were enrolled into the study. These patients attended the Integrated Counseling and Testing Centres (ICTC) and outpatient clinics under LEPRA Society Hyderabad, India. Inclusion and exclusion criteria were as follows: HIV patients in the age group 18–60 yrs, with CD4+ cell counts were >350 cells/mm^3^ were considered eligible for recruitment. Those with a history of tuberculosis, ART/ ATT, other opportunistic infections, chronic illnesses were considered ineligible.

#### 2.1.2 Healthy volunteers

Hundred healthy HIV sero-negative volunteers in the age group 18–60 yrs were recruited as controls. Healthy controls had no history of TB or ATT. Those with diabetes, autoimmune diseases and any other immunosuppressive conditions were excluded from the study.

This study was approved by Institutional Review Board of Blue Peter Public Health Research Centre, Hyderabad, India. Written and informed consent was obtained from all the participants enrolled in the study. Demographic details of the participants including age, gender, history of Bacilli-Calmette-Guerin (BCG) vaccination, history of pulmonary TB and treatment profiles were collected and shown in Table 1. Participants were categorised into LTBI+ and LTBI- using an in-house IGRA, irrespective of their HIV status and designated as HIV+LTBI+, HIV+LTBI- and HIV-LTBI+, HIV-LTBI- respectively.

**Table 1:** Demographic and clinical characteristics of study subjects.

	HIV-LTBI-	HIV-LTBI+	HIV+LTBI-	HIV+LTBI+
Number of participants	50	50	50	50
**Mean CD4 counts**	NA	NA	452	552
**Mean years of onset of HIV infection**	NA	NA	5.1	5.4
**Mean age**	33	34	34	35
**Percentage of males and females**	43, 57	44,56	29,71	35, 65
**BCG scar percentage**	85	87	71	60
**History of TB infection**	No	No	No	No
**ART treatment**	NA	NA	No	No

NA = Not Applicable, HIV-LTBI-: HIV negative healthy individuals without latent TB infection, HIV-LTBI+: HIV negative healthy individuals with latent TB infection. HIV+LTBI-: HIV patients without latent TB, HIV+LTBI+: HIV patients with latent TB.

### 2.2 Antibodies and other reagents

For flow cytometry, we used anti-CD3 FITC (BioLegend, USA, Cat#300440) and PerCP (BD Biosciences, USA, Cat#347344), anti-CD56 FITC (BioLegend, USA, Cat#318304) and APC (BD Biosciences, USA, Cat#555518), anti-CD16 PE (BioLegend, USA, Cat#302008); for staining memory cells anti-KLRG1 FITC (BioLegend, USA, Cat#138410), anti-CD27 PE (BD Biosciences, USA, Cat#555441); for intracellular staining anti-IL-32α (R&D Systems, USA, Cat#IC30402A) and anti-IFN-γ APC (BioLegend, USA, Cat#502512) antibodies were used. Magnetic beads conjugated to anti -CD56 (Miltenyi Biotec, Germany, Cat# 120-000-307) and -CD14 antibodies (Miltenyi Biotec, Germany, Cat# 120-000-305) were used for positive selection of NK cells and monocytes respectively.

For *in vitro* stimulation assays, we used ESAT-6 (BEI Resources, USA, Cat#NR-50711) and CFP-10 (BEI Resources, USA, Cat#NR-50712) peptide pools consisting of 21 and 22 individual peptides belonging to 6-kDa ESAT-6 and 10-kDa CFP-10 respectively. γ-irradiated Mtb *H37Rv* whole cells were used for some experiments (BEI resources, USA, Cat#49098).

### 2.3 Isolation and culture of PBMCs

PBMCs were isolated by density gradient centrifugation using ficoll-hypaque (Sigma-Aldrich, USA, Cat#10771). Briefly, whole blood diluted with RPMI-1640 (Sigma-Aldrich, USA, Cat#R5886), was layered over an equal volume of ficoll and centrifuged for 30–40 minutes at 2000 RPM without brakes. PBMC layer was collected, cells were washed twice, and enumerated by trypan blue staining. 2 x10^6^ cells/well were then cultured in 24-well plates in RPMI 1640 medium containing 1% penicillin/streptomycin (Sigma-Aldrich, USA, Cat#P4333), L-Glutamine (Sigma-Aldrich, USA, Cat# G7513) and 10% heat-inactivated human AB serum, with or without γ-*Mtb*, CFP-10 and ESAT-6 (10 μg/ml each). Cells were cultured at 37°C in a humidified incubator with 5% CO_2_ atmosphere. After 96 hours, cell-free culture supernatants were collected, aliquoted and stored at −80°C until cytokine and chemokine concentrations were measured.

### 2.4 Flow cytometry

Percentages of CD16, CD27 and KLRG1 on CD3^-^ CD56^+^ cells were determined by flow cytometry. Freshly isolated and cultured PBMCs, were surface stained with anti-CD3, anti-CD56 and anti-CD27 in respective tubes and incubated in the dark at 4°C for 30 mins. Cells were then washed twice and suspended in 1x fix-perm buffer (eBiosciences Inc, CA, USA) for permeabilization. After incubation for 30 mins cells were washed twice in 1x permeabilization wash solution. Anti- IL-32α and anti- IFN-γ were then added to respective tubes and re-suspended in staining buffer. After incubation at 4°C for 30 mins, cells were washed in PBS with 2% FCS and fixed in 1% paraformaldehyde (Sigma-Aldrich, USA, Cat#P6148) before acquisition and analysis on FACSCalibur (BD Biosciences, USA).

### 2.5 Gating

From the gated total lymphocyte population, NK cells were identified by surface expression of CD16, CD56 and absence of CD3. Memory like NK cells were determined by gating on CD3^-^CD56^+^ cells expressing CD27, KLRG1. IFN-γ and IL-32α expressing NK cells were gated on CD3^-^CD56^+^CD27^+^ from total lymphocyte population. All the cell subsets were mentioned as a percentage of gated population. Representative flow cytometry plots are shown in S1 and S2 Figs.

### 2.6 Determination of latent TB infection in the study subjects

LTBI was determined according to our previous published protocols. Briefly, 2 x 10^6^ of freshly isolated PBMCs were stimulated with and without 10μg/mL each of CFP-10 and ESAT-6 antigens, incubated at 37°C for 96 hrs. IFN-γ released by PBMCs was measured using commercial human interferon-gamma kit (eBioscience Inc., San Diego, CA, Cat# 88-7316-86) following manufacturer instructions. IFN-γ concentration was calculated using MPM software version 6.1. Subjects were categorized into LTBI- and LTBI+ depending on the IFN-γ value.

### 2.7 Isolation of CD56+ and CD14+ cells

PBMCs were isolated by differential centrifugation over Ficoll-Paque as mentioned in the previous section. To the cell pellet, undiluted magnetic beads conjugated to anti-CD56 were added. NK cells were isolated by positive immune-magnetic selection. To the NK cell depleted cell pellet, undiluted anti-CD14^+^ magnetic beads were added to isolate monocytes by positive immune-magnetic selection. These positively selected cells were >95% pure as measured by flow cytometry.

### 2.8 Culture of NK cells and autologous monocytes with γ-irradiated Mtb H37Rv

Freshly isolated CD56^+^ and CD14^+^ cells (2 x 10^6^ CD56^+^ with 2 x 10^5^ CD14^+^) were cultured in 12-well plates in 10:1 ratio in RPMI 1640 medium containing 1% penicillin/streptomycin, L-Glutamine and 10% heat-inactivated human serum. 10μg/mL γ-irradiated Mtb H37Rv cells were added to some wells for stimulation. Unstimulated wells served as controls. Cells were incubated at 37°C in humidified 5% CO_2_ atmosphere for 72 hrs. After termination, cells were stored in trizol (Sigma-Aldrich, USA, Cat#T9424) cell-free supernatants were aliquoted and stored at −80°C until cytokine concentrations were measured.

### 2.9 Measurement of granzyme B, IFN-γ, IL-15 and CCL5 (RANTES) in cell culture supernatants and plasma

Granzyme B, IFN-γ and IL-15 in the culture supernatants were measured by ELISA (ThermoFisher Scientific, USA, Cat**#** BMS2027, Cat#88-7620-88 and Cat# 88-7316-86 respectively) following manufacturer’s instructions. Cytometric Bead Array CBA kit (BD Biosciences, San Diego, CA) was used to quantify CCL5 (BD Biosciences, USA, Cat#564752) in culture supernatants. 50 μl of serial diluted standards or individual samples were added to respective tubes with pre-mixed capture beads specific for CCL5 chemokine. 50 μl of PE-labeled secondary detection antibody was added to all the tubes. After incubation in dark for 3hrs at 4°C, tubes were washed to remove any unbound antibody from the cell suspension. Pellet was resuspended and samples were then acquired on FACS calibur with cell-quest pro software. CCL5 concentration in samples were calculated using CBA software (BD Biosciences, San Diego, CA).

### 2.10 Statistical analysis

Results are shown as median and IQR. Kruskal-Wallis test was used to determine significances between two or more groups. p<0.05 was considered statistically significant. Comparison within groups was performed by *t* test, as appropriate. Pearson correlation analysis was performed to examine the relationship between NK cell subsets and cytokine production using Graph pad prism v6.0 software. Results from correlation analysis are presented as S3 Fig.

## 3. Results

### 3.1 NK cell subsets in HIV- and HIV+ individuals with and without LTBI

**Fig 1:**
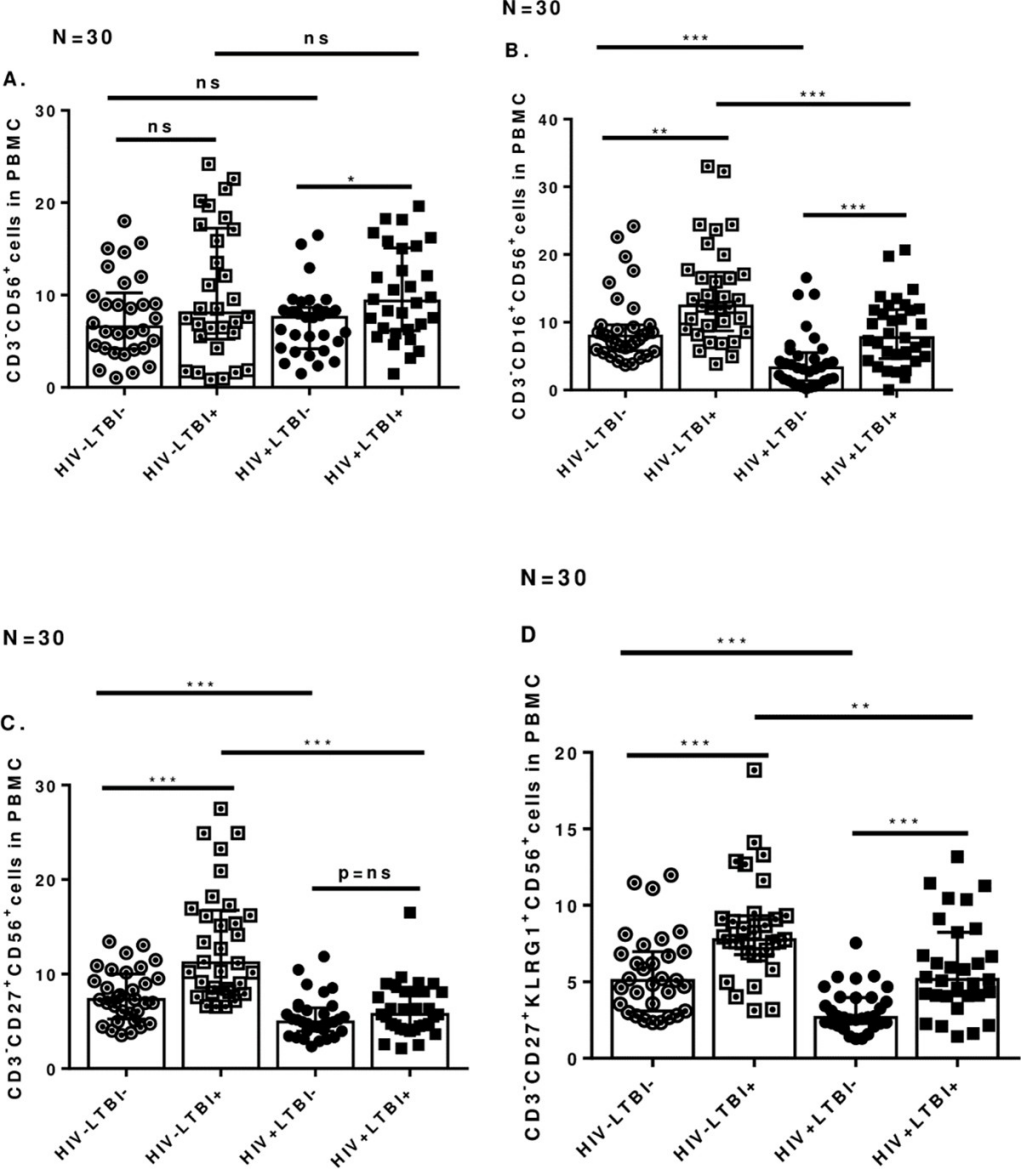
NK cell subpopulations in fresh PBMC. Freshly isolated PBMC from HIV-LTBI-, HIV-LTBI+ individuals, HIV+LTBI- and HIV+LTBI+ patients (30 donors in each group) were stained with Abs to CD3, CD56, CD16, CD27 and KLRG1. Flow cytometry was used to measure the percentages of **A.** CD3^-^CD56^+^ **B.** CD16^+^CD56^+^ **C.** CD3^-^CD27^+^CD56^+^ and **D.** CD3^-^ CD27^+^KLRG1^+^CD56^+^cells. P values across the groups were derived using Kruskal Wallis One-way ANOVA and unpaired T test. Data is shown as median and interquartile range. Boxes show the median and interquartile range, and whiskers show the 5th and 95th percentile values. ns denotes not significant, * denotes p < 0.05, ** denote p < 0.01, *** denote p < 0.001, **** denote p < 0.0001.

In freshly isolated PBMCs, percentages of CD3^-^CD56^+^ cells were similar in LTBI+ individuals with or without HIV (Fig 1A). Percentages of CD3^-^CD56^+^CD16^+^ cells however, were higher in HIV-LTBI+ than HIV-LTBI- individuals (Fig 1B, p = 0.002) and HIV+LTBI+ patients (Fig 1B, p = 0.0005); also in HIV-LTBI- individuals compared to HIV+LTBI- patients (Fig 1B, p<0.0001).

Percentages of CD3^-^CD27^+^CD56 and CD3^-^CD27^+^KLRG1^+^CD56 cells were higher in HIV-LTBI+ compared to HIV-LTBI- individuals (Fig 1C, p = 0.0001, Fig 1D, p = 0.004 respectively), HIV+LTBI+ patients (Fig 1C, p<0.0001, Fig 1D, p = 0.002 respectively); and in HIV-LTBI- individuals compared to HIV+LTBI- patients (Fig 1C, p = 0.0005, Fig 1D, p = 0.002). HIV+LTBI- patients had lower CD3^-^CD27^+^KLRG1^+^CD56 cells compared to HIV-LTBI- (p<0.0001) and HIV+LTBI+ (p<0.0001) patients.

### 3.2 Defective expansion of memory like NK cells in PBMCs from HIV+LTBI+ patients

**Fig 2:**
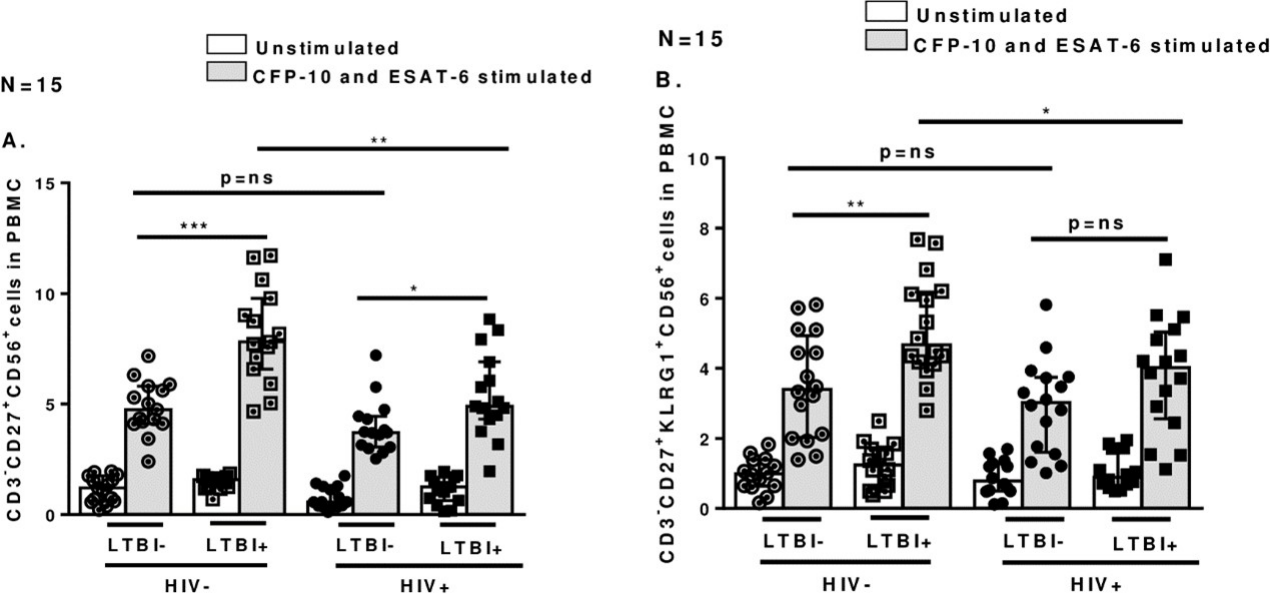
Memory like NK cell expansion in HIV+ and HIV- individuals without and with latent tuberculosis infection in response to Mtb CFP-10 and ESAT-6. PBMCs from HIV-LTBI-, HIV-LTBI+ individuals, HIV+LTBI- and HIV+LTBI+ patients (15 donors in each group) were isolated and cultured with and without Mtb CFP-10 and ESAT-6 (10 μg/ml each). After 96 h, percentages of **A**.CD3^-^CD27^+^CD56^+^ and **B.** CD3^-^ CD27^+^KLRG1^+^CD56^+^cells were determined by flow cytometry. P values across the groups were derived using Kruskal Wallis One-way ANOVA and unpaired T test. Data is shown as median and interquartile range. Boxes show the median and interquartile range, and whiskers show the 5th and 95th percentile values. ns denotes not significant, * denotes p < 0.05, ** denote p < 0.01, *** denote p < 0.001, **** denote p < 0.0001.

In our previous studies we demonstrated that memory like NK cells (CD3^-^CD56^+^CD27^+^KLRG1) expand in LTBI+ individuals in response to Mtb antigens ESAT-6 and CFP-10, but not in LTBI- individuals [10]. In this study we determined if HIV infection has any effect on the expansion of memory like NK cells in HIV+LTBI+ individuals in response to Mtb antigens. We cultured fresh PBMC isolated from HIV- and HIV+ individuals with and without LTBI with Mtb antigens ESAT-6 and CFP-10 as mentioned in methods section. After 96 hours, percentages of CD3^-^CD27^+^CD56 and CD3^-^CD27^+^KLRG1^+^CD56 cells were determined by flowcytometery. We observed that PBMCs from HIV+LTBI+ patients had significantly low memory like NK cell expansion compared to HIV-LTBI+ individuals (Fig 2A, p = 0.001 and Fig 2B, p = 0.02).

### 3.3 Memory like NK cells in PBMCs from HIV+LTBI+ patients produce low IL-32α and IFN-γ

**Fig 3:**
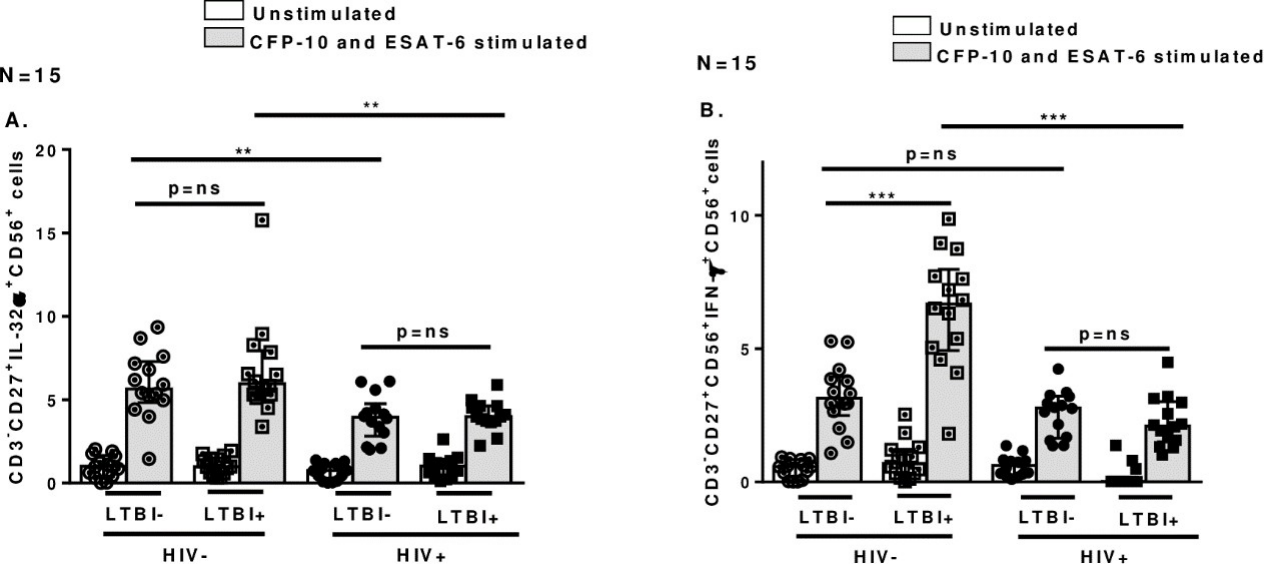
Cytokine production by memory-like NK cells in response to CFP-10 and ESAT-6. PBMCs from HIV-LTBI-, HIV-LTBI+ individuals, HIV+LTBI- and HIV+LTBI+ patients (15 donors in each group) were isolated and cultured with and without CFP-10 and ESAT-6 (10 μg/ml each). After 96 h, cells were permeabilised and intracellular **A**. IL-32α and **B**. IFN-γ production by memory like NK cells was determined by flow cytometry. P values across the groups were derived using Kruskal Wallis One-way ANOVA and unpaired T test. Data is shown as median and interquartile range. Boxes show the median and interquartile range, and whiskers show the 5th and 95th percentile values. ns denotes not significant, * denotes p < 0.05, ** denote p < 0.01, *** denote p < 0.001, **** denote p < 0.0001.

NK cells are the major producers of IFN-γ during early stages of infection and the role of IFN-γ during Mtb infection is well known [20]. IL-32α is another recently discovered pro-inflammatory cytokine produced by NK cells [21]. After identifying that HIV+LTBI+ individuals had defective memory like NK cell expansion, we determined if there was also an impaired cytokine production by these cells. PBMC were cultured, IL-32α and IFN-γ producing CD3^-^CD56^+^CD27^+^ cells were determined by flow-cytometery. As shown in Fig 3, in respose to ESAT-6 and CFP-10, PBMCs from HIV+LTBI+ patients had low expansion of CD3^-^CD56^+^CD27^+^IL-32α^+^ (Fig 3A, p = 0.002) and CD3^-^CD56^+^CD27^+^IFN-γ^+^ (Fig 3B, p<0.0001) cells compared to HIV-LTBI+ individuals.

### 3.4 Suboptimal NK cell and monocyte interactions in PBMCs isolated from HIV+LTBI+ patients

**Fig 4:**
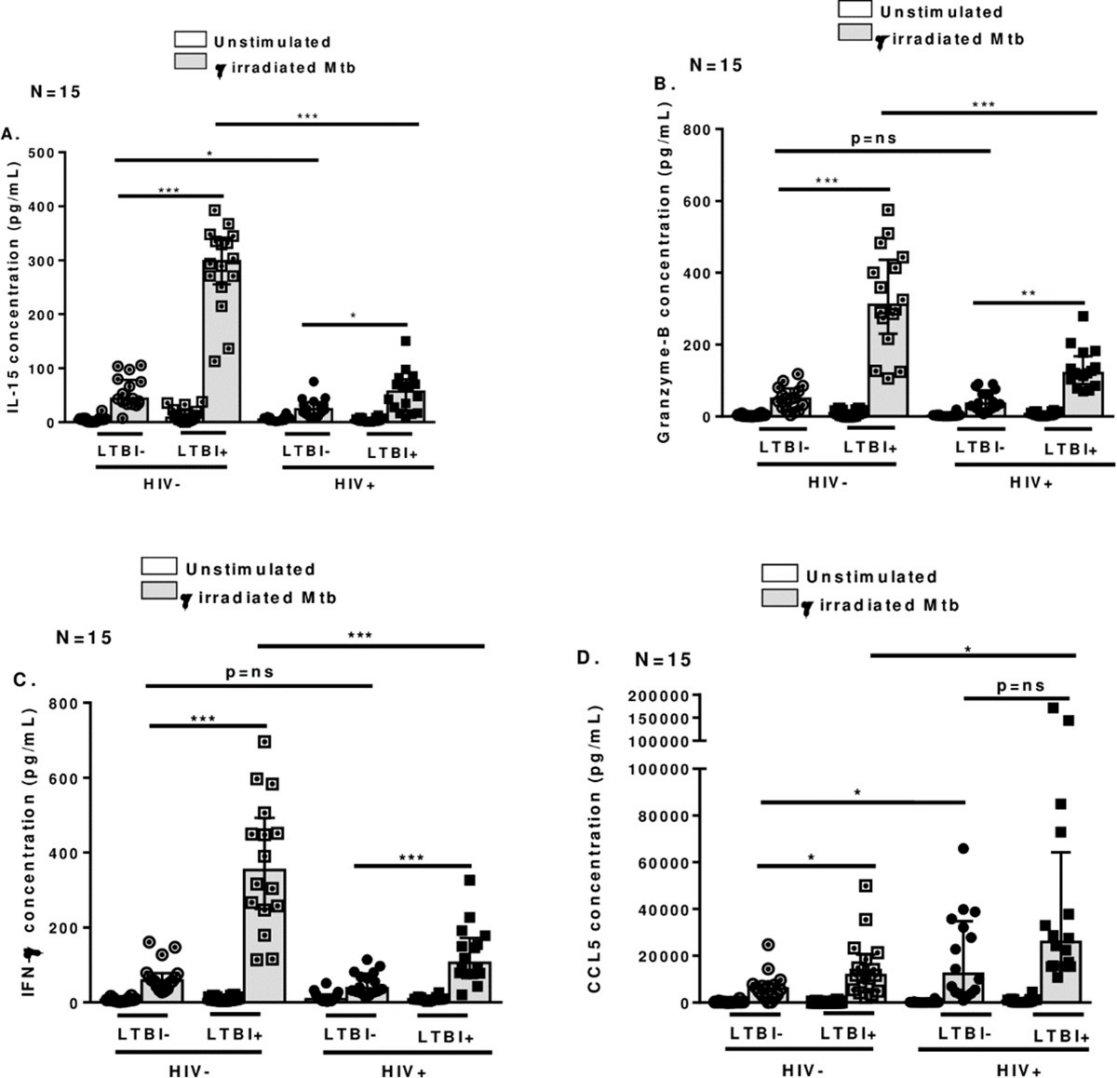
Cytokine and chemokine production by Mtb cultured NK cells and monocytes. CD56^+^ and CD14^+^ cells from PBMCs from HIV-LTBI-, HIV-LTBI+ individuals, HIV+LTBI- and HIV+LTBI+ patients (15 donors in each group) were isolated by magnetic selection and cultured with or without γ-irradiated Mtb H37Rv (10 μg/ml). After 72 h, **A.** IL-15, **B**. CCL5, **C**. Granzyme-B and **D.** IFN-γ levels in culture supernatants were determined by ELISA. P values across the groups were derived using Kruskal Wallis One-way ANOVA and unpaired T test. Data is shown as median and interquartile range. Boxes show the median and interquartile range, and whiskers show the 5th and 95th percentile values. ns denotes not significant, * denotes p < 0.05, ** denote p < 0.01, *** denote p < 0.001, **** denote p < 0.0001.

Interactions between antigen presenting cells like dendritic cells and NK cells are vital for optimal immune responses against pathogens and cancers [22]. IL-15 is essential for the activation of NK to release cytotoxic granules and for production of IFN-γ during various intracellular infections [23]. Similarly, CCL5 plays an important role in the infiltration of NK cells to the site of infection and augments NK cell cytotoxicity and IFN-γ production [24]. We determined whether reduced functional activity of NK cells in HIV+LTBI+ patients was due to suboptimal NK cell and monocyte interactions. We isolated NK cells from PBMCs of HIV-LTBI+ individuals and HIV+LTBI+ patients as mentioned in methods section and cultured them with autologous monocytes with or without γ-irradiated Mtb H37Rv. After 72 hours, we measured IL-15, CCL5, granzyme B and IFN-γ levels in culture supernatants. In response to γ-irradiated Mtb H37Rv, NK cells from HIV+LTBI+ patients produced significantly low IL-15 (p<0.0001, Fig 4A), granzyme B (p<0.0001, Fig 4B) and IFN-γ (p<0.0001, Fig 4C) but high CCL5 (p<0.04, Fig 4D) compared to HIV-LTBI+ individuals.

### 3.5 Frequencies of KLRG1+NK cell subsets correlate with IL-15 and granzyme production in HIV patients

To identify the relationship between frequency of antigen specific NK cell subpopulations and cytokine production, correlation analysis was performed. A positive correlation was observed between antigen specific CD3^-^CD27^+^KLRG1^+^CD56 cells and IL-15 production in HIV+LTBI+ patients (p = 0.04, r = 0.5) while no significant correlation with IFN-γ, CCL5 or granzyme was observed (S3D Fig). Similarly, a positive correlation was observed between antigen specific CD3^-^CD27^+^KLRG1^+^CD56 cells and granzyme production in HIV+LTBI- patients (p = 0.04, r = 0.05) while no significant relation was observed with IL-15, IFN-γ and CCL5 production (S3C Fig). No correlation was observed between NK cell subsets and cytokine expression in HIV- individuals with or without LTBI (S3A and S3B Fig).

## 4. Discussion

Natural killer (NK) cells are critical in first-line defense of the immune system contributing to immunity against viral infections, but information on their role in defense against intracellular bacteria is limited [4, 5]. Previously, we and others found that NK cells mediated immune responses play an important role against Mtb infection [25, 26]. We also found that memory like NK cells (CD3-NKp46+CD27+) expand and provide protection against Mtb infection in BCG vaccinated mice [10, 11]. In this study we observed defective expansion of memory like NK cells in response to Mtb antigens in LTBI+ individuals with HIV. Defect in NK cell expansion was also associated with production of low IFN-γ, IL-32α and Granzyme B compared to HIV- individuals with LTBI. In response to Mtb stimulation NK cells cultured with autologous monocytes, produced low IL-15, granzyme but enhanced CCL5 production in HIV+ patients compared to HIV- individuals with LTBI. HIV patients have dysfunctional memory like NK cells, weak cytokine production and cytolytic activity [27, 28]. Our study for the first time demonstrates that HIV infection impairs protective memory like NK cell mediated immune responses against Mtb infection in LTBI+ individuals.

We found low NK cells in HIV patients, indicated by a decrease in NK cell numbers in the circulation as well as in response to Mtb stimulation. HIV infection induces pathological redistribution of frequencies, phenotypes and functions of NK cell subsets affecting overall NK cell anti-viral activity. HIV infection particularly decreased CD3^-^CD56^+^CD16^+^ and CD3^-^CD56^+^CD27^+^ NK cell subsets which are potent producers of cytokines to counteract viral replication and progression of latent TB infection. Expression of CD27 on NK cells promotes long-term survival of functional effector memory cells in HIV patients with latent TB. CD27^+^ expression on NK cells promotes interactions with T cells and defective CD27 expression in HIV patients suggests defective interaction between NK cells and B and/or T cells.

NK cells and epithelial cells are the major source for IL-32 [29]. IL-32 enhances TNFα, IL-8, IL-6, IL-1β, and various chemokine production through activation of NF-kB pathway in monocytes and macrophages. IL-32 enhances NK cell cytotoxicity and is used to improve the efficiency of NK cell-based immunotherapy against cancers [30, 31]. During Mtb infection, IL-32 was associated with differentiation of monocytes into dendritic cells with increased capacity to present antigens to CD8+ T cells. IL-15 and IFN-γ induce IL-32 which inturn enhances the expression of antimicrobial peptides defensin and cathelicidin within monocytes [32]. The essential role of IFN-γ during Mtb infection is already known [33, 34]. We found memory like NK cells from HIV+LTBI+ patients produced low IFN-γ and IL-32 in response to Mtb. Contrary to our findings, it was demonstrated that IL-32 contributes to early control of HIV replication, but counteracts longterm immunity to HIV by interfering with cell activation, proliferation, damping antiviral immune responses favoring HIV persistence [35]. Further determination of mechanism/s involved in reduced IL-32 and IFN-γ may help in developing novel therapies to treat and prevent TB in HIV+ patients.

NK and dendritic (DC) cell interactions promote the development of adaptive immune responses [36]. NK cells also modulate DC function and maturation [36, 37]. NK-mediated killing of HIV infected-DCs is a crucial event required for early elimination of infected target cells [38]. NK-DC interactions are altered during HIV infection [39]. In the current study we demonstrated that there is a suboptimal NK and monocyte interactions leading to reduced IL-15, granzyme B and IFN-γ production in HIV+ patients with LTBI. Mice with impaired IL-15 production were associated with defective CD8 and NK cell effector functions and subsequent administration of IL-15 enhanced CD8 T-cell number and survival [40, 41]. IL-15 is crucial for production, differentiation and survival of antigen-specific CD8 T cells [42]. IL-15 also enhances expression of effector NK cell molecules like perforin, granzymes A, B and IFN-γ production by CD8 T cells [43]. IL-15 also directly activates and enhances NK cell survival and proliferation and IFN-γ production of HIV-specific CD8 T and NK cells [43, 44]. Our study demonstrates that HIV infection alters reciprocal interactions between Mtb cultured NK cells and monocytes that lead to dysfunctional effector functions by memory like NK cells and monocytes.

IL-15 and IL-15R are important for survival and expansion of antigen specific NK cells, the mechanisms of differentiation into memory phenotype following viral infections are not clearly understood. Following murine CMV(MCMV) infection, 30% of NK cells express a killer cell lectin-like receptor G1 (KLRG1) which differentiates cells into memory phenotype [45, 46]. It was shown that IL-15 differentially regulates KLRG1^+^ cells and treatment with IL-15 increased accumulation of KLRG1^+^ mature NK cells [47, 48]. Our correlation analysis also provided some insight into the relation between KLRG1^+^ NK cells and cytokine production in HIV patients. We observed a positive correlation between antigen specific CD3^-^CD27^+^KLRG1^+^CD56 cells and IL-15 production in HIV+ patients with latent TB. We hypothesize that the defective IL-15 production in response to Mtb stimulation can be associated with low KLRG1^+^CD56 cells in HIV patients. In LTBI negative HIV patients however, a positive correlation between KLRG1^+^ NK cells and granzyme production was observed. It was interesting that this correlation between NK cells and effector molecules was observed only in HIV patients and not healthy controls. Our study highlights the importance of expansion of memory NK cells and secretion of effector molecule in control of latent TB infection especially in HIV patients. We speculate that low expansion of KLRG1^+^ NK cells by HIV patients could be a reason for defective IL-15 and granzyme production in response to Mtb stimulation.

CCL5 is one of the key chemokines, that regulate migration of NK cells to the site of infection [46]. CCL5 also increases the cytotoxicity and promote release of cytotoxic granules by NK cells [49, 50]. During Mtb infection, CCL5 regulates and activates the recruitment of macrophages, NK cells and T-cells to the site of infection to limit bacterial growth by establishing granulomas. In our previous study, we showed that the concentration of CCL5, was lower at the site of TB infection compared to plasma [51]. It was shown that HIV proteins enhance CCL5 in patients with chronic infection which causes excess inflammation [52, 53]. HIV patients have been shown to have elevated levels of CCL3 and CCL5 in serum compared to healthy controls. CCL5 along with MIP-1α and MIP-1β were shown to increase the viral replication in PBMCs [54]. Binding of GP120 protein of HIV-1 to CCR5, or CXCR4, is a primary step of the viral entry to T cells. Elevated expression of CCL5 was reported during HIV-TB coinfection, however the exact role of this chemokine during LTBI is not explored widely. In the current study, we found impaired IL-32, IFN-γ and granzyme B production by NK cells of HIV+LTBI+ individuals despite high CCL5 production. Our findings further suggest high CCL5 production by HIV+ patients with LTBI enhanced viral replication and activation of T cells resulting in altered response to antigen and defective cytokine production and signalling pathways in NK cells of HIV+LTBI+ patients. This increase in CCL5 plays a more prominent role in HIV pathogenesis by increasing immune activation and permissibility than its antiviral function.

In summary, we found that HIV infection in LTBI+ individuals interferes with optimal NK cell and monocyte interactions leading to defective cytokine production, expansion and function of memory like NK cells. HIV infection inhibits the release of IFN-γ, IL-15 and there by IL-32 production by NK cells. KLRG1^+^ NK cell expansion, which is crucial for cytokine production and effective functioning of NK cells and was also impaired in HIV+LTBI+ patients. Further understanding of these mechanisms will facilitate development of interventions to prevent progression of LTBI in HIV infected individuals.

## 5. Conclusion

We analysed the changes in phenotypic and functional profiles of NK cells in HIV infected individuals with LTBI. Association between expansion of memory like NK cells in latent TB and HIV was highlighted. We conclude that HIV infection not only disrupts NK cell mediated elimination of infected cells, but also production of potent cytokines essential for control of Mtb. Understanding mechanisms for expansion of memory-like NK cells during HIV and latent TB facilitate development of interventions to prevent progression of LTBI to TB in high risk population.

## Supporting information

S1 Fig A representative flow cytometry plot showing gating strategies. A representative flow cytometry plot for Fig 2A and 2B are shown. Plots (A), (B) show Total lymphocytes; (C), (D) show CD3^-^CD56^+^ cells and (E), (F) show CD3^-^CD27^+^KLRG1^+^CD56^+^ cells in HIV- and HIV+ individuals respectively.
(DOCX)

S2 Fig A representative flow cytometry plot showing gating strategies. A representative flow cytometry plot for Fig 3A and 3B are shown. Plots (A), (C) show CD3^-^CD27^+^ IL-32α^+^CD56^+^ cells and (B), (D) show CD3^-^CD27^+^ IFNγ^+^CD56^+^ cells in HIV- and HIV+ individuals respectively.
(DOCX)

S3 Fig Correlation analysis between NK cell subsets and cytokine production. Pearson correlation analysis was performed using Graphpad prism v6.0 software to examine the relationship between NK cell subsets and cytokine production. Plots indicate relationship between (A) HIV-LTBI- (B) HIV-LTBI+ (C) HIV+LTBI- (D) HIV+LTBI+ individuals respectively.
(DOCX)

## Abbreviations

Mtb: *Mycobacterium tuberculosis*
HIV: Human immunodeficiency virus
TB: Tuberculosis
NK: Natural killer
LTBI: Latent Tuberculosis Infection
CFP-10: Culture Filtrate Protein
ESAT: Early Secreted Antigenic Target
KLRG1: Killer cell lectin-like receptor subfamily G member 1
IFN: Interferon
IL: Interleukin
ART: Anti-retroviral therapy
ATT: Anti-tuberculosis treatment
FITC: Fluorescein isothiocyanate
PE: Phycoerythrin
PerCP: Peridinin-chlorophyll-protein Complex
APC: Allophycocyanin
PBS: Phosphate buffered saline
FCS: Fetal calf serum
PBMC: Peripheral Blood Mononuclear Cells
ELISA: Enzyme Linked Immuno-Sorbent Assay
SD: Standard deviation
SE: Standard error
CCL5: Chemokine (C-C motif) ligand 5
RANTES: Regulated on Activation
Normal: T Expressed and Secreted
