# Supplementary figures and images for: Defective expansion and function of memory like natural killer cells in HIV+ individuals with latent tuberculosis infection
Source: PLoS One. 2021 Sep 13;16(9):e0257185. doi: 10.1371/journal.pone.0257185 (PMC8437280; doi:10.1371/journal.pone.0257185)

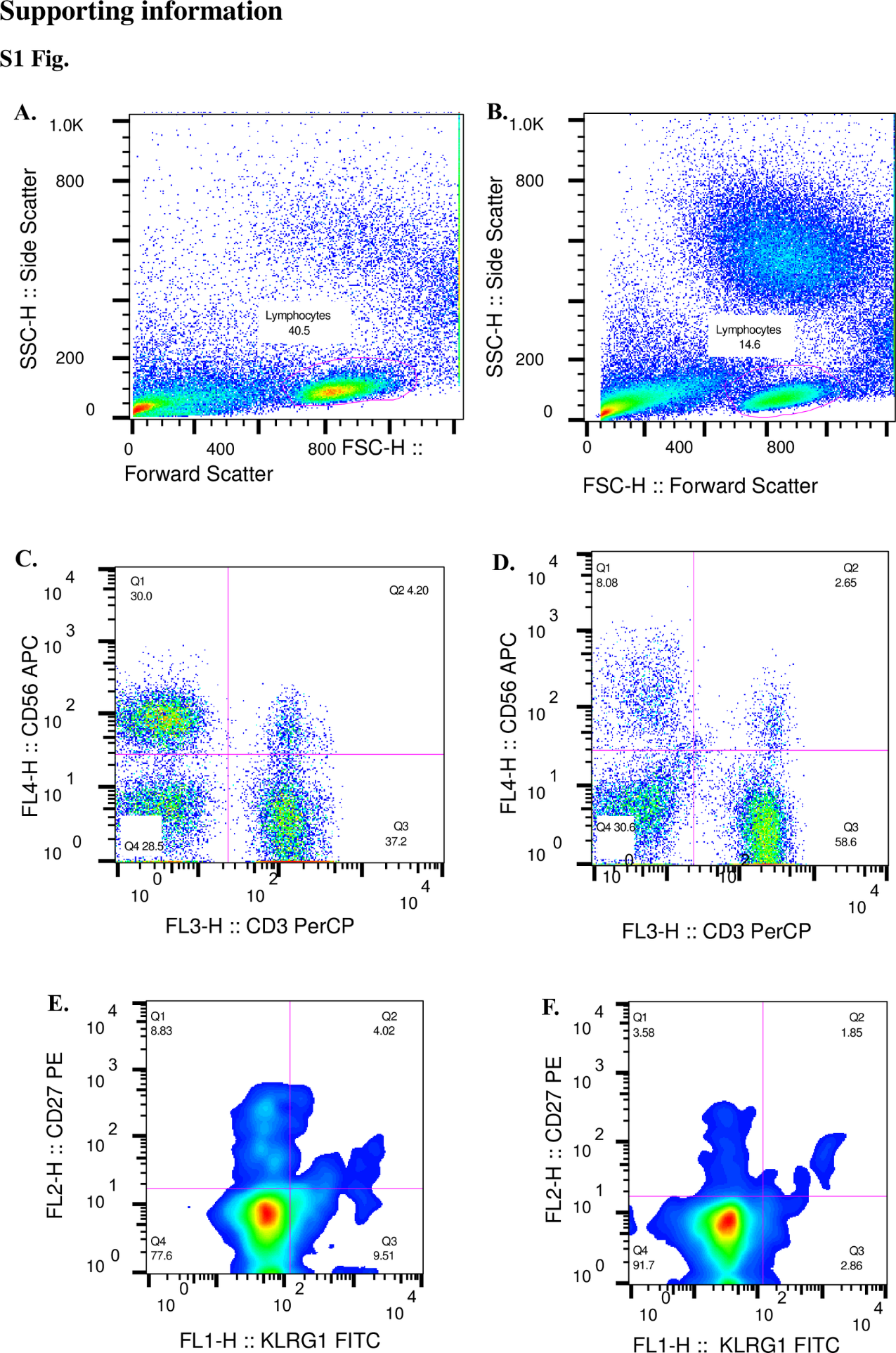

Supplement: S1 Fig — A representative flow cytometry plot for Fig 2A and 2B are shown. Plots (A), (B) show Total lymphocytes; (C), (D) show CD3-CD56+ cells and (E), (F) show CD3-CD27+KLRG1+CD56+ cells in HIV- and HIV+ individuals respectively. (DOCX) [file pone.0257185.s001.docx]

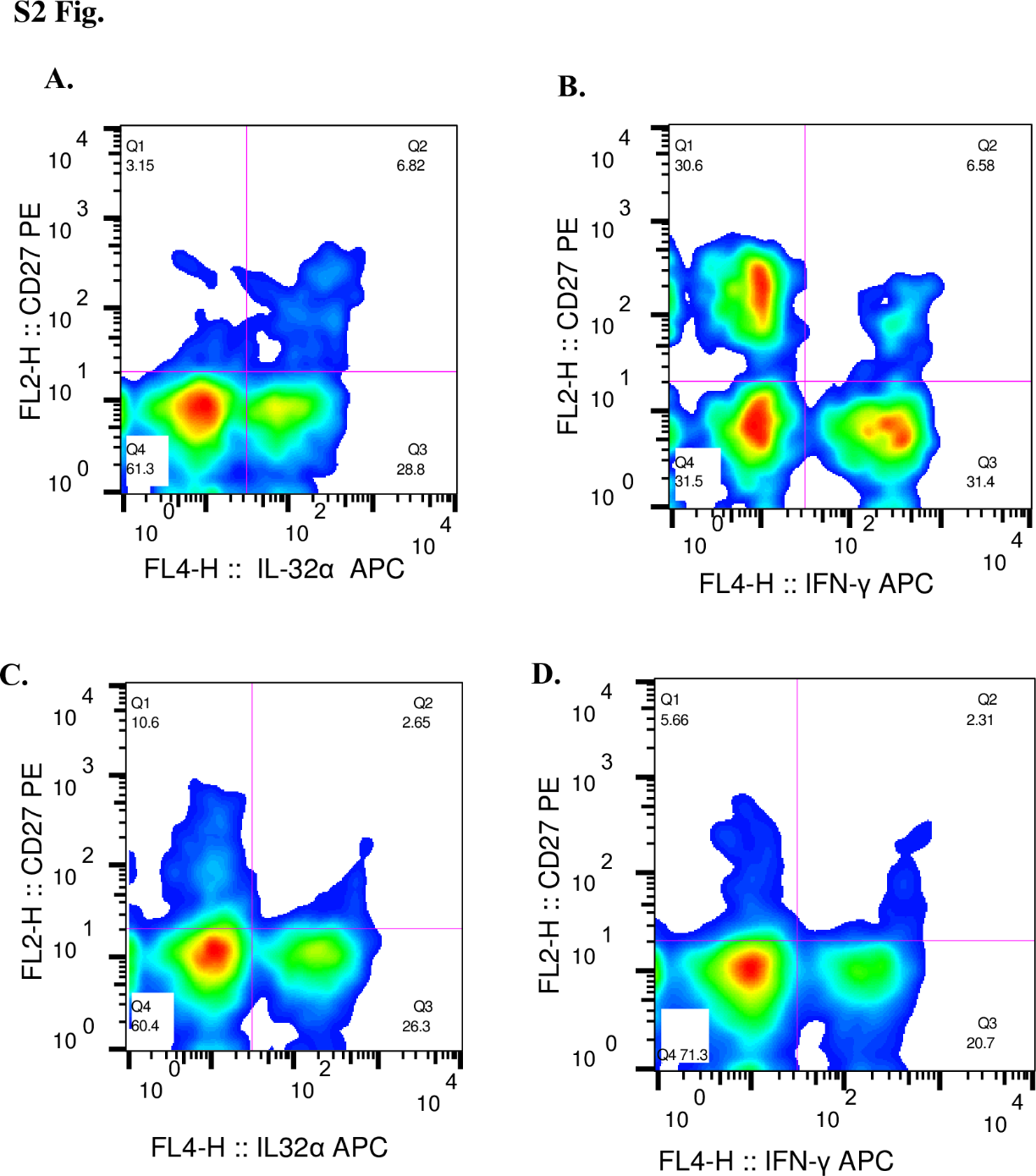

Supplement: S2 Fig — A representative flow cytometry plot for Fig 3A and 3B are shown. Plots (A), (C) show CD3-CD27+ IL-32α+CD56+ cells and (B), (D) show CD3-CD27+ IFNγ+CD56+ cells in HIV- and HIV+ individuals respectively. (DOCX) [file pone.0257185.s002.docx]
