# Supplementary material for: Defective expansion and function of memory like natural killer cells in HIV+ individuals with latent tuberculosis infection
Source: PLoS One. 2021 Sep 13;16(9):e0257185. doi: 10.1371/journal.pone.0257185 (PMC8437280; doi:10.1371/journal.pone.0257185)
Supplement: Supplementary file 2 [file pone.0257185.s003.docx]

S3 Fig.

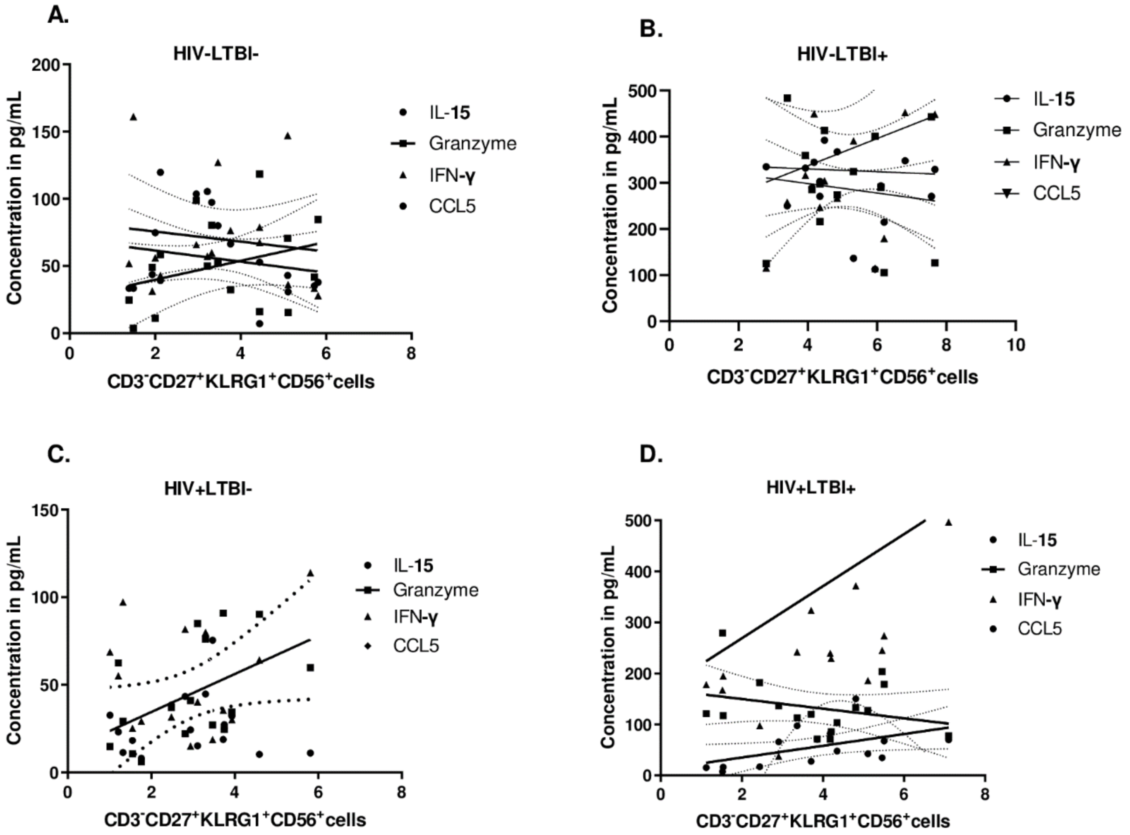
A.
HIV-LTBI-
Concentration in pg/mL
CD3⁻CD27⁺KLRG1⁺CD56⁺cells
IL-15
Granzyme
IFN-γ
CCL5
B.
HIV-LTBI+
Concentration in pg/mL
CD3⁻CD27⁺KLRG1⁺CD56⁺cells
IL-15
Granzyme
IFN-γ
CCL5
C.
HIV+LTBI-
Concentration in pg/mL
CD3⁻CD27⁺KLRG1⁺CD56⁺cells
IL-15
Granzyme
IFN-γ
CCL5
D.
HIV+LTBI+
Concentration in pg/mL
CD3⁻CD27⁺KLRG1⁺CD56⁺cells
IL-15
Granzyme
IFN-γ
CCL5
